# Geosystems‘ Pathways to the Future of Sustainability

**DOI:** 10.1038/s41598-019-50937-z

**Published:** 2019-10-08

**Authors:** Mihai Voda, Shadrack Kithiia, Edward Jackiewicz, Qingyun Du, Constantin Adrian Sarpe

**Affiliations:** 1 Dimitrie Cantemir University, 3-5 Bodoni Sandor st., 540545 Targu Mures, Romania; 2 Department of Geography and Environmental Studies, University of Nairobi, Nairobi, 30197-00100 Kenya; 3 Geography and Environmental Studies, California State University, Northridge, CA 91330-8249 USA; 4 School of Resource and Environmental Science, Wuhan University, 129 Luoyu Road, 430079 Wuhan, China; 5 Romanian National Waters Administration, 33 Samuel Koteles st., 540057 Targu Mures, Romania

**Keywords:** Socioeconomic scenarios, Sustainability

## Abstract

The world’s future development depends on effective human-computer linkages. From local to global, the virtual illustrations of a geographical place have to emphasize in an integrative approach peoples‘ key position in the Geosystem. Human values and social networks are now empowered by the unlimited creativity of smartphone applications. Our Geosystem grounded theory envisions that the sustainable management of natural resources is a lifelong learning environment where the poor communities have access to the new technological advances. This paper will attempt to show the effectiveness of Geomedia techniques in the Geosystems identification, evaluation, and valorization processes for the benefit of local inhabitants. This present research methodology uses smartphone apps, Google Earth environmental datasets, Global Positioning Systems, and WebGIS for a geographical investigation and objective assessment of regions throughout the world. The results demonstrate that self-sustainable Geosystems will always be capable to regulate, control and assess progress towards their dynamic equilibrium state, continuously adapting to environmental and societal changes.

## Introduction

This article investigates why and how we as humans need to redefine our position in the Geosystem. The environmental factors and their natural interactions are considerably influenced by advances in modern technology. The study suggests that human attributes have to be valorized to maintain the balance of natural elements, to improve the harmony of a geographical space and to assess the progress of all Geosystem components^1,2^. Importantly, it outlines that in the new technological era, where Big Data and smartphone applications manipulate the world, we have to ensure and promote lifelong learning opportunities for all, especially for the poor traditional communities where the specific myths and legends went afar a *meme*, being transformed in particular egregores, influencing the collective thoughts^3–6^. The development of our young people social and emotional abilities is integrated in the technological era, given the amount of time spent using high-tech devices. Therefore it is a sine qua non condition for the future Geosystems complex problem-solving strategies.

Smartphone’s geographic data and satellite imagery are offering placement opportunities for any virtual portrayal of the Geosystem’s functional components. For example, the isolated indigenous communities can geotag illustrations of their unique Geosystem’s natural state and validate discrepancies of aerial images, when needed^7,8^. Observing and documenting environmental changes such as forest loss could mitigate illegal logging and subsequently, potential flood damage in the future^9^. Furthermore, the cultural unit assessments considerably improve the compatibility of these Geosystem technologies and local knowledge. Sharing the information to the communal network authorities generates overflow coefficients, adjustment, better prognosis, promoting transparency and public awareness^10^. Big Data and Artificial Intelligence have to increase the Geosystems collaborative actions and social cohesion for the environment protection and cultural ecosystem services development, helping people to detect genuine answers for the difficult issues that exist in our developing complex world^11^.

This innovative approach examines interaction between global, social and human networks facilitating the analysis of entire Geosystems where the digital revolution is empowering the place-based activities extension for the wellbeing of all inhabitants. Ten years ago, traditional community members would have spent considerable amounts of time on webpage design and search engine connectivity to attract international tourists, but today’s Airbnb platform makes it possible for any cosy home with hospitable sharing people to be on display instantaneously for free^8,12^. This research shows that more localized indigenous Geosystems have higher resilience and adaptability because they have a strong egregore and access to technology. They also promote lifelong learning opportunities for all and are a part of functional transformative networks. This approach reflects the most important features for the critical functionality of the analysed individual Geosystems. Shabanova (2017)^13^ analysed methods of energy use in different environments, emphasizing the egregore subtle potential and efficiency.

This study is based on a decade of personal observations and research into dynamic Geosystems around the Globe, including: Romanian region of Transylvania; Monument Valley in the US; the highlands of Bolivia, Peru, Kenya and India; the Greek islands of Thassos and Lefkada; Indonesian islands of Bali and Java; the Ulrike Mountains; the sheer cliffs of Hua Shan; Bukhara; and ancient Cairo.

Our research proves that the integrative development of Geosystems is not occurring in isolation but as functional constituents of transformative networks. According to Ravasz & Barabasi (2003)^14^ in many social networks, ‘the scale–free topology and high clustering coexist‘. The inner quintessence of each Geosystem, represented by the people’s collective group mind, its egregore, significantly contributes to their wellbeing. The Austrian small town of Worlg experiment from the midst of the Great Depression, proved the importance of social connections and collaborative actions for a community’s sustainable development^15^.

After summarizing previous studies on Geosystems’ applications and their connection to sustainability, the data and methodology are described, the results examined and the main findings and implications are discussed. The future of sustainability is relying on human analytical thinking capabilities to adapt and shape the Geosystem changes. Ryan^16^ argues that people can create their own future with the modern virtual and augmented reality opportunities. But the new technologies will never contribute enough to a sustainable future if they are not correlated to the peoples‘ collective values and social skills expansion. According to Croft (2018)^17^, more resilient supply networks will generate welfare for local communities if the inhabitants’ access to the new technologies and continuing education is granted.

An assessment will be first made on the efficiency of the functional Geosystems, on a localized individual basis, and then the efficacy of the new technologies for the Geosystems egregore preservation, development and integration in transformative networks will be examined.

## Research Methodology

This research is based on the Geosystem grounded theory using Geomedia techniques to integrate all relevant environmental components into virtual functional illustrations of the analyzed geographical spaces. The individual Geosystems internal relations and unique attributes were synthetized and processed in correlation with individual visual and mental imagery constructions. The structure-element interaction and the Geosystem’s specific feature characteristics were determined. Then, the more localized Geosystems behavioral computations revealed their resilience and adaptation in critical functionality conditions^18–20^.

With this innovative approach, the investigation of geographical space gains new dimensions in terms of Geosystem’s attribute identification, performance evaluation and the valorization of unique characteristics for its quality extension and extrapolation.

The data collection process was performed between November 2013 and April 2018 by visiting all sites. Specific detailed input data was gathered during research field trips using smartphone geo-applications and direct on-site observations. The Geosystems‘ principal components analysis, elements correlation and unique attributes evaluation were operated on geographical informational systems such as EarthExplorer, LandsatLook Viewer and Google Earth using Landsat 8 satellite imagery. The Application Programming Interfaces tools were used for the identified Geosystem’s factors in-depth analysis and social media data processing. Extensive investigation of the identified attributes displayed the results of their functionality inside the Geosystem^8,9,21^. The Geomedia techniques comprise all the new high tech solutions and smart applications that are facilitating the creation and online distribution of images, symbols, maps and text, geolocationally integrated by digital systems, initiated and preserved by the social networks exchange capabilities.

Easily apprehended Geomedia techniques were developed with local community members in Romania, Kenya, Bolivia, India, and Indonesia. Google Earth datasets of Sarmisegetuza Regia area, Ogiek community forests, Yungas region, Garhwal Himalaya and Merapi Volcano were used to explore, visualize and analyze the local Geosystems of each area. The profile graphs were constructed with 3D Analyst from ArcGIS 10 after Garmin GPS on field validation of the selected tracks^9,22,23^.

The geo-referenced illustrations of chosen sites from the Monument Valley, Machu Picchu and Hua Shan Mountains were processed using Geomedia techniques and certified on social media channels^9,21,24^. Then, the assessment of the validated imagery was performed for every location, comparing the unique attributes virtual representations in WebGIS. Extracting the most pertinent Geosystems‘ illustration, the common physical characteristics were identified in the islands of Lefkada, Thassos, Bali, and Java while similar cultural features were revealed in the old Bukhara and Cairo cities^25–27^.

The application of the invert geo-coding methodology allowed the semantic connotation disclosure of certain geo-sites such as Ogiek Community in Kenya, the Sphynx rock in Romania and the Ulrike Mountain near Bergen, in Norway. The smartphone images with geo-location were analyzed and the interchange information digital data extracted for the virtual Geosystems‘ map creation^8,28,29^.

Data was collected for the 2013–2018 period from publicly available Geomedia information on Google Search, Facebook and Airbnb platforms. Landsat 8 satellite imagery, EarthExplorer, LandsatLook Viewer and Google Earth environmental datasets were used to identify changes, evaluate trends and quantify dissimilarities in the Geosystems‘ analysis. For each location different numbers of geottaged posts were identified and analyzed. Geo-data was gathered on field with mobile GPS (Sygic, Garmin) and smartphone apps (Maverick, Google Photos, GTCamera) to validate the tracks information and geo-referenced illustrations (Table 1). A total number of 1937 km of tracks were assessed with 3D Analyst from ArcGIS 10 after Garmin GPS on field validation for each Geosystem. 750 geo-tagged posts and 1580 geo-referenced illustrations were analyzed and the interchange information digital data extracted for the virtual Geosystems evaluation. The Geomedia Analytic Hierarchy (GAH) was used to obtain the Unique Attributes score, the Innovation diffusion importance and the Geosystem effectiveness values (<2 very weak; 3–4 weak; 5–6 moderate; 7–8 strong; 9–10 very strong). Every Geosystem has unique attributes generated by the geographical places‘ features, history, local traditions and customs. The innovation diffusion value is reflecting the Geomedia’s role in facilitating innovation and transformative networks integration. The Geosystem’s effectiveness reveals the attributes functionality inside each analyzed geographical location. The Arrow’s theorem^30^ was applied to validate the results obtained with Geomedia techniques and GAH processes, providing evidence that local communities’ access to the new technological advances can help them learn how to balance the matter, energy and information fluxes, to identify and promote their Geosystem’s valuable assets in order to protect their living environments and increase their livelihoods.

**Table 1:** Geosystems‘ behavioral computation.

Country	Geosystem name	Google search results	Unique attributes score (GAH)	Facebook geotagged posts	Airbnb platform places	Innovation diffusion (GAH)	Geo-referenced illustrations	On field validated tracks (km)	Geosystem effectivness	Arrow points	Final Arrow score	Geosystem effectivness/Arrow score differences
Greece	Lefkada Island	6530000	10	46	300+	10	110	120	10	7	10	0
Indonesia	Jimbaran	8400000	9	23	300+	9	60	120	10	6	9	1
Greece	Thassos Island	5390000	8	41	300+	10	90	130	10	6	9	1
Peru	Machu Pichu	25500000	10	23	300+	8	70	148	9	6	9	0
China	Hua Shan	74200000	10	22	114	8	50	28	10	5	8	2
Romania	Bran Castle	4710000	10	38	111	10	76	40	10	5	8	2
Taiwan	Taiwan	1280000	9	34	300+	10	38	86	10	5	8	2
United States	Alabama Hills	102000000	10	29	19	8	36	78	9	5	8	1
Romania	Sphynx	57600	9	34	125	8	69	18	8	5	8	0
United States	Monument Valley	125000000	10	28	23	8	83	59	10	6	9	1
India	Hemkund Sahib	266000	8	29	26	8	72	52	8	4	7	1
Indonesia	Yogyakarta	189000000	7	22	300+	8	29	80	9	4	7	2
United States	Antelope Canyon	22100000	8	36	212	9	16	18	9	4	7	2
Indonesia	Batur Volcano	799000	8	31	148	8	28	140	9	4	7	2
Bolivia	Death Road	17400000	10	8	23	4	102	128	8	4	7	1
Uzbekistan	Bukhara	8380000	8	27	72	8	86	53	10	5	8	2
Kenya	Hell`s Gate	7380000	9	11	202	4	26	71	9	4	7	2
India	Jama Masjid	21200000	8	28	70	8	27	6	8	3	6	2
Romania	Viscri	339000	9	41	18	10	34	56	9	4	7	2
Egipt	Old Cairo	74900000	8	19	33	6	58	31	6	2	5	1
Kenya	Mau Forest	264000	8	18	12	6	62	145	6	2	5	1
Romania	Borgo Pass	169000	9	15	6	6	48	64	5	2	5	0
China	Terracota Army	7700000	9	15	60	5	70	12	7	3	6	1
Norway	Ulrike Mountain	261000	7	8	300+	4	64	52	7	3	6	1
Indonesia	Merapi Volcano	1690000	9	25	55	8	52	84	8	4	7	1
Romania	Saschiz	272000	3	31	5	4	16	12	6	1	4	2
Romania	Bagaciu	82000	2	28	4	2	28	32	4	1	4	0
Austria	Worlg	245000	9	18	85	8	56	60	9	4	7	2
Romania	Sarmisegetuza Regia	33000	7	22	23	5	24	14	3	0	3	0

Several iterations of coding were operated to validate the accuracy of data collected from 419 semi-structured interviews conducted between November 2013 and April 2018 with local communities’ representants, NGO managers, Airbnb hosts, tourism operators and potential home stay providers. 231 people from 18–39 years of age and 187 over age 40 responded to our questions in twenty two Geosystems around the globe (Table 2).

**Table 2:** Geosystems‘ adaptation to changes capacity.

Country	Geosystem name	Total respondents	General acces to Geomedia	People aged 18–39 years	Geomedia use competence (18–39)	People over 40 years old	Geomedia use competence (>40)	Adaptation to changes capacity
Greece	Lefkada Island	20	0.294	11	0.25	9	0.163	7.07
Greece	Thassos Island	17	0.265	10	0.25	7	0.184	6.99
Peru	Machu Pichu	19	0.265	11	0.2	8	0.143	6.08
China	Hua Shan	18	0.265	12	0.2	6	0.163	6.28
Romania	Bran Castle	25	0.294	14	0.25	11	0.184	7.28
Taiwan	Taiwan	19	0.294	9	0.25	10	0.204	7.48
United States	Alabama Hills	14	0.265	5	0.222	9	0.163	6.5
Romania	Sphynx	22	0.265	11	0.222	11	0.143	6.3
United States	Monument Valley	15	0.265	4	0.222	11	0.163	6.5
India	Hemkund Sahib	21	0.206	15	0.222	6	0.163	5.91
Indonesia	Yogyakarta	20	0.265	13	0.5	7	0.184	9.49
United States	Antelope Canyon	14	0.265	7	0.5	7	0.163	9.28
Bolivia	Death Road	21	0.265	13	0.175	8	0.143	5.83
Uzbekistan	Bukhara	24	0.206	15	0.222	9	0.122	5.5
Romania	Viscri	22	0.265	11	0.222	11	0.163	6.5
Egipt	Old Cairo	23	0.206	13	0.2	10	0.122	5.28
Kenya	Mau Forest	18	0.206	10	0.15	8	0.102	4.58
Romania	Borgo Pass	19	0.235	11	0.15	8	0.102	4.87
Indonesia	Merapi Volcano	16	0.265	9	0.2	7	0.143	6.08
Romania	Saschiz	13	0.206	7	0.15	6	0.082	4.38
Romania	Bagaciu	23	0.206	12	0.175	11	0.082	4.63
Romania	Sarmisegetuza Regia	16	0.235	9	0.15	7	0.082	4.67

Some of the questions asked in the semi-structured interviews (‘How would you characterize your community’s collective mind, the local egregore?‘, What is your competence level in Geomedia use?‘, How would you value the Geomedia access in your Geosystem’s area?‘) required supplementary explanations involving rich answers from the community members.

## Geosystem’s Unique Functional Attributes

The analysis identified a power law generated by the social networks interference with the indigenous communities. The extension of the mobile networks geographical coverage and the access to the smartphone technology facilitates the extrapolation of indigenous collective values. Furthermore, the Geosystem’s principal components analysis revealed the beneficial influence of the information flux input for the communities’ collaborative actions.

This article is the first to consider the Geosystem’s grounded theory in the context of modern technology advances. The smartphone phenomena are continuously affecting communities’ life. Social networks such as Facebook and WhatsApp are driving communication and information exchanges beyond imagination. Currently, the Google Maps and Google Earth applications are offering enhanced opportunities for the geographical space visualization and analysis. Moreover, Airbnb is transcending tourism development, enabling creativity, garnering and sharing community values and allowing visitors to share their travel experiences^7,20^.

The study finds evidence for the relevant role of unique attributes in the process of geographical location image creation and their cultural representations in accordance to the visitors‘ mental perceptions and classification^31–34^. A first example is the Borgo Pass area, perceived as central for Bram Stoker’s Dracula castle location but peripheral within the Romanian Dracula network, where Brasov with Bran Castle retain the central position^8^. The second is the *Death Road* from Bolivia, where the wisdom of the crowd and the social networks generate an attractive image of the Cordillera Real Mountains, creatively combining Incan culture with the dangerousness attribute^20^.

**Figure 1:**
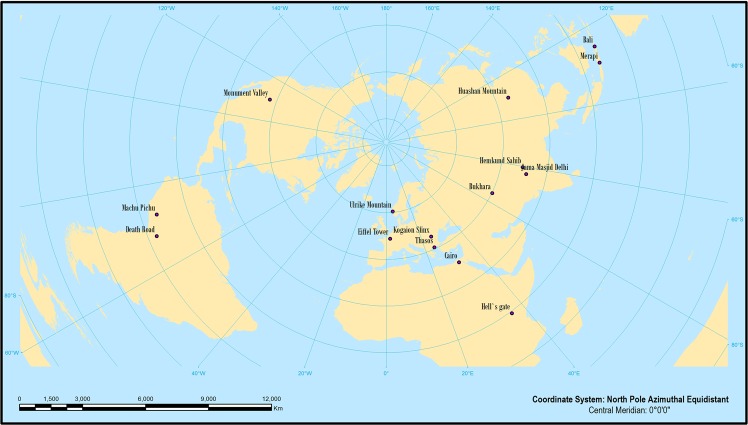
The analyzed Geosystems in North Pole Azimuthal Equidistant projection.

The analysis reveals that the geotagged photos became the visual representation of all analyzed geographical location (Fig. 1), contributing to the construction of people’s *naïve images* if there is at least one reference to that Geosystem’s unique attribute^24,35^. For example, Paris’s stereotypical image contains the Eiffel Tower, San Francisco has the Golden Gate Bridge, Peru has the ancient Incan Machu Picchu and Transylvania has Dracula in the international collective image^8,36^. It is important to notice the *postcard effect* extrapolation possibilities through the smartphone social network applications. Today’s technological advancements enable communities to build their own future^16^. As the Geomedia techniques are increasingly assimilated by most of the society members, it is worth exploring the Geosystem’s collective images creation opportunities. Currently, the Old Bukhara city geographical space constitutes a unitary illustration of the local Geosystem ancient history, reflected in the well-preserved buildings of the Silk Road shining pearl. This unique attribute communal visualization as stepping back in time was created by the Uzbekistan people with the support of local authorities and visionary policy makers, who learned that an integrative approach to the sustainable development dynamics will attract many foreign visitors in search for new and unique experiences. The common heritage of the Silk Road spiritual occurrence was incorporated in the local community’s traditions.

**Figure 2:**
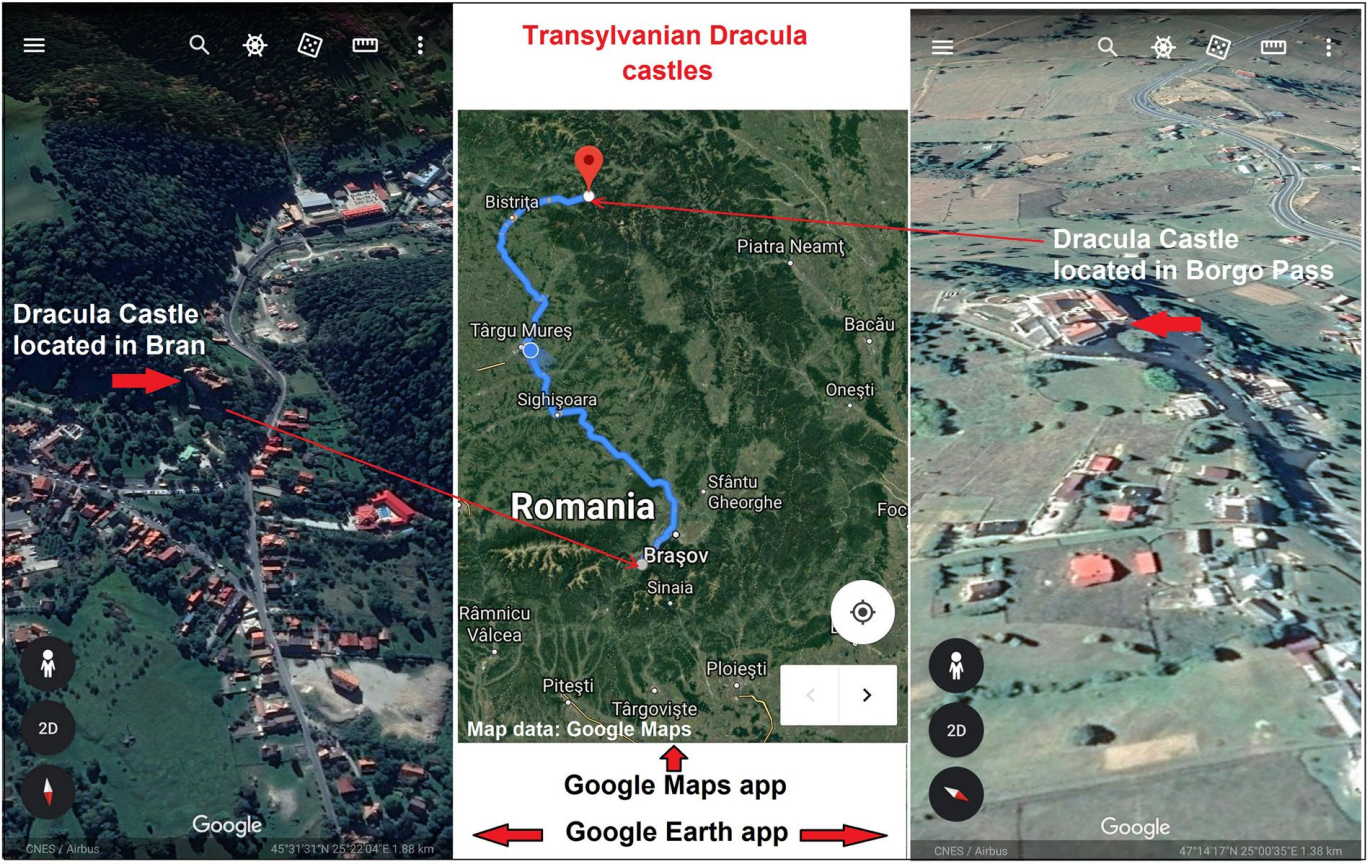
Smartphone apps illustration of Transylvanian Dracula locations^23,39^.

Transylvania’s Dracula (Fig. 2), Bolivia’s Death Road and Uzbekistan’s Bukhara related Geosystems are balanced in a dynamic equilibrium state. Their egregore structured people attitude to development issues, affecting their comprehension in a positive way^6^. Today’s technological advances can revitalize and promote any traditions through various smartphone applications as long as they are still kept alive by the natives^7^. The constant energy infusions from the international visitors are driving the indigenous Geosystems‘ flux, regulated and controlled by the thriving local communities. Their involvement constitutes an essential condition for the sustainable tourism development success in many parts of the world once considered backward or underdeveloped^37,38^.

This study suggests that authentic geographical locations such as Monument Valley, Machu Picchu, Hua Shan or Death Road (*El Camino de la Muerte*) could represent sustainability models for similar Geosystems. For example, the Alabama Hills in the Sierra Nevada Mountains, a common filming location for *Western* movies, has the Navajo tribe’s Monument Valley functional attributes reference. The indigenous people Geosystem’s dynamic provides cultural ecosystem services through horse trails and the Navajo community dirt road network. Delaforge (1987)^40^ considered that an egregore is a collective group mind generated when people purposefully gather and act for a common goal. Hwang and Stewart (2017)^41^ argue that the local inhabitants’ collective initiatives are founding the community-based structures sustainability. Moreover, the American *Wild West* stereotypical image has its best reflection in the Monument Valley. Romania’s Sarmisegetuza Regia, Bucegi’s Sphynx ancient areas or Hemkund Sahib have the Machu Picchu, Hua Shan or *El Camino de la Muerte* development examples. The Ogiek’s invisible inseparable fusion with the Mau forest complex is a further illustration of mental thinking and environment geosystems sustainability in a global dimension. The selected geographical locations’ common attributes are represented by the mountainous landscapes and the ancient cultures specific marks observed in the local Geosystems. The unique attributes assessment revealed considerable asset similarities but utility discrepancies in terms of sustainable tourism valorization. The Hemkund Sahib Pilgrimage phenomenon, for example, was definitely contributing to the local communities livelihoods increase in the past years and raising the environmental education level in the same time^7^.

Coined by Dawkins (1976)^42^, the memes constitute a ‘viral phenomenon‘ that can naturally grow inside a Geosystem which is viewed as a cultural entity. The meme is self- organized to survive, constantly adapting to its environmental changes. Furthermore, our study observations around the world, suggests that it can replicate itself and even transform its Geosystem to sustain its expansion^3^.

The analysis shows that the Geomedia visualization techniques used in advanced Geosystems to promote the local community assets are easily validated by the incoming visitors which are sharing them with the world on social networks, contributing to the destination collective stereotypical image consolidation^20^. Although the Machu Picchu Geosystem’s rather inconvenient location in the Peruvian mountains questions its functionality and construction purposes, the site is continuously visited and the indigenous communities thrive. Considerably far from Cuzco, the Inca’s culture pearl is reachable after passing high mountain ranges and crossing dense tropical forest in the Amazon river basin^36^. The modern tourist infrastructure with railway and gravel roads significantly contributes to the Machu Picchu Geosystem dynamics, facilitating the inbound tourist flux administered by the local community in collaboration with the regional authorities in an integrative approach. The unique functional attributes, the construction myths and legends can always constitute tourism attraction themes^43^.

Taiwan Island represents one of the most balanced Geosystems in terms of dynamic equilibrium between the matter, energy and information fluxes. The analysis suggests that advantageous sites were chosen for human settlements and considerable value assigned to the indigenous organization in order to emphasize the local geographical space unique identities^44,45^. The mountain forests are sustainably managed by the local communities and forest guard members who are protecting them against the ‘mountain rats‘. The old Alishan railway from Chiayi to Tashan that was constructed under the Japanese occupation for the red cypress (*Chamaecyparis formosensis*) lumbering was originally transformed in one of the best eco-touristic routes^46,47^. The destination image was built based on the old Chinese folk song ‘Kao San Chin‘ (Green High Mountain) related to the unique qualities of the local people in Alishan mountainous area: girls beautiful like crystalline water and boys strong as the mountain rocks. The cultural phenomenon is smartly regulated by the Taiwanese authorities.

**Figure 3:**
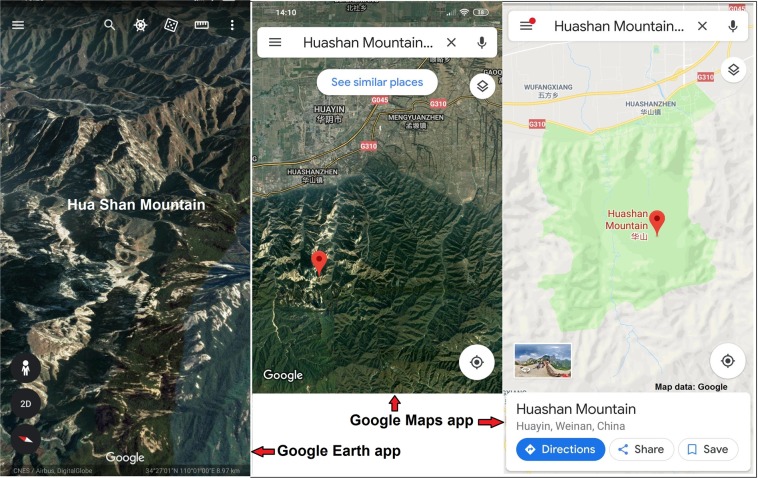
Hua Shan unique attributes illustration^23,39^.

In China, the Hua Shan Mountain temples spectacular landscape and religiously significant Geosystem (Fig. 3) is effectively correlated with the nearby ancient and mysterious Terracotta Army site^48^. The Chinese governmental policy is encouraging the rural communities’ active involvement, oversight and control by the local administration. In Bergen, the Ulrike Mountain access trails are commonly used during week-ends by the local Norwegians, being a part of their indigenous culture and a good leisure activity for international visitors. Furthermore, tourism development can generate revenue for community members, contributing to improved management of the Geosystem’s resources^8^.

The Death Road (*El Camino de la Muerte*) Geosystem in Bolivia is located in the Cordillera Real Mountains, connecting the La Paz area highlands to the Amazon River basin and its natural resources, especially the *coca* leaves, used for the mine workers. The local Coroico community benefits from the tourists’ constant input into the Geosystem, who in turn pay around 100 USD for a mountain bike experience on the 64 km long descending route from 4470 m glacial lakes to 1000 m rain forest.

The research revealed that in Arizona, the Antelope Canyon sustainable tourism expansion significantly contributed to the Page area community development. The social media promotion of the natural gorges attracted more visitors. The Chinese tourists’ inbound phenomenon determined accommodation system extension and an increase in prices from the previous summer season. Valorizing similar geographical features, the Hell’s Gate Geosystem from Kenya has a balanced dynamic in terms of energy input but lacks in funding for local communities, being focused mainly on wild game protection, managed by the Kenyan Wildlife Service while on the other hand the Maasai cultural artifacts, dress and culture has remained a significant tourist attraction (as printed in many postcards).

## Geosystem’s Sustainable Development Solutions

The Google Maps smartphone application, Maverick tracking and Sygic offline GPS systems were used for the selected geographical locations correlation analysis in order to identify and evaluate the indigenous Geosystems‘ common and unique functional attributes.

The islands of Lefkada, Thassos, Bali and Java have unique cultural heritages and exquisite geographical features. The sacred inland mountains are associated with ancient Gods, still worshiped by the local communities. The limestone sheer cliffs, the volcanic rocks and the emerald waters illustrate some of the world’s most beautiful beaches^25,27,49^. While the European islands benefit from the mainland proximity and the access to the new technological advances, the Far East Indonesian islands are still struggling for development solutions. Unlike the balanced Geosystems of Lefkada and Thassos where the Airbnb phenomenon is widely spread between Greek inhabitants, the Javanese and Balinese Geosystems has the system developed mainly in Yogyjakarta and Jimbaran areas. Facing environmental carrying capacity problems in Merapi and Batur Volcano areas, the Indonesian indigenous communities have to learn how to create the future self-sustainable Geosystems where the new technologies will help them regulate and control the inbound tourists and four-wheel drive vehicles number^8^.

Romania’s Sarmisegetuza Regia and Busteni’s Sphynx in the Carpathian region and India’s ancient areas of Hemkund Sahib and Badrinath both surrounded by the majestic peaks of the Himalayan mountains have the same functional attributes represented by the common geographical features and their spiritual significance for indigenous people^50–53^. The Romanian governmental authorities are restricting public access into the mountainous areas based on the European Union biodiversity policy although there is no apparent adverse effect of roads on the richness of local habitats^54,55^. Voda *et al*.^9^ analyzed the illegal logging sites correlation with the mountain roads network, emphasizing the new technology role for the Wild Carpathia forest’s protection. The cultural ecosystem services are important for the sustainable use of any mountainous Geosystem, as it has been proven in Peru’s Machu Picchu or China’s Hua Shan sacred peaks, where the psychological entity created by the local people awareness is intolerant to dissent^6,36,48^.

**Figure 4:**
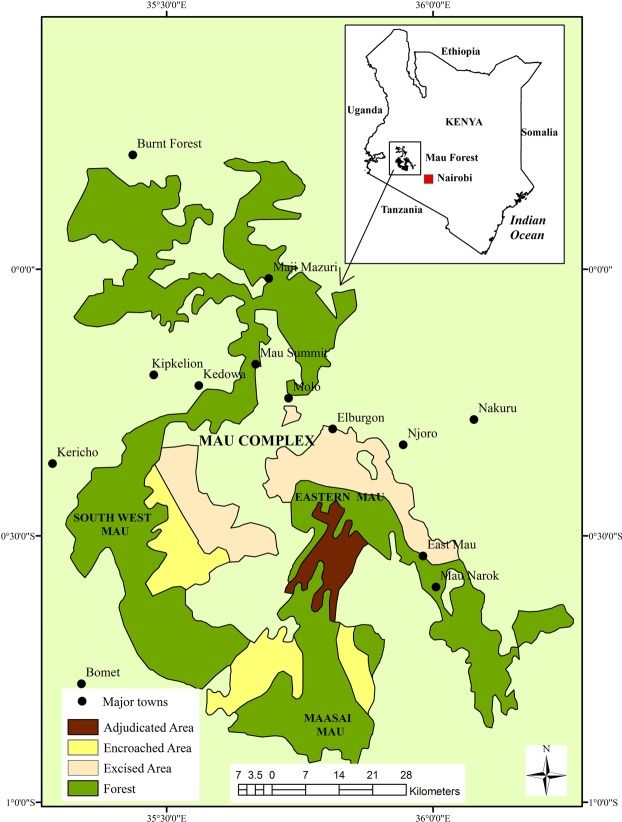
Ogiek’s community Mau Geosystem.

On the other hand, it has been noted that the Ogiek Community was among the first people to settle in the East African forests, among them the Mau Forest complex in Kenya^56^. This led to the development of an extricable relationship with forests which were used as sacred sites for beehives, prayers, hunting and gathering as well as for ancestral graves. It is argued that the Ogiek attach spiritual, emotional and economic values to the Mau Forest as they have historically relied on it for food, shelter, and identity^57^. However, with the advent of British colonization of Kenya, the Ogiek, were evicted from the East Mau Forest complex, with the intention of assimilating them with the Kalenjin and the Maasai who had both been evicted from the forest and settled on the reserves. The colonial policy was informed by the need to settle white settlers on the best land available, at the expense of the Kenyan communities^56,58^. The Ogiek’s still harbor the belief that sustainable use of the Mau complex forest will rely heavily on their resettlement and coexistence with forest resources (Fig. 4). They argue that they have coexisted with the forest in an historically sustainable manner^59^. Currently, the Ogiek’s have been settled a few kilometers from the forest without or with limited access to the forest complex. They believe that they can provide a solution to the forest degradation if allowed to go back to the forest through use of their local community guards. During the study visits, the Ogiek’s were found to be knowledgeable in the use of smartphones and hence can easily map out their resources. It should be noted that after the forest incisions of the 1980’s, the Mau Forest complex experienced the highest rates of degradation due to charcoal burning, timber harvesting and agricultural cultivation^60^. From field work research and interviews conducted in and around the Mau forest complex it was evident that these were the key factors that contributed to the forest destruction. The research also confirmed that members of the Ogiek community were conversant and competent in the use of Geomedia platforms. They are using the methods in collaboration with Kenya Wildlife Service (KWS) in monitoring in forest encroachment and enforcing conservation efforts through their local rangers stationed inside the forest.

The common attributes of the Old Cairo Geosystem were identified and compared to the Old Bukhara city features. The cultural richness is reflected in the buildings architectural style, indigenous religious traditions and local artisans’ products^61,62^. But there were no registered tourists in the narrow streets sector situated behind the Sultan Hassan Mosque. After assessing the Geosystem’s functionality we came to the conclusion that using the Geomedia techniques, the locals can easily create a distinctive self-sustainable tourist site. As the Old Bukhara’s imagery developed in correlation with the Silk Road and the 1001 stories, Old Cairo can use the new technological advances to increase its virtual representation qualities. Today’s reality reflects an ancient place that looks more like the area behind Jama Masjid Mosque towards Delhi’s Railway Station, with a stuck-in-time ambience.

Today’s geographical data availability substantially facilitates the Geosystem’s unique attributes identification and assessment. The system elements moving patterns were established from analysis in correlation with the Geosystem’s functional properties and unique attributes. This study’s geographical locations were chosen based on their Geosystem’s specific behavioral computation. The research revealed the indigenous people essential role in the protection and preservation processes of their living environments. The locals’ history, legends and myths confer authenticity to the geographical place^43^. Communities’ access to the new technological advances can help them learn how to promote the Geosystem’s valuable assets and increase their livelihoods^8^.

## Results Discussion

It is generally agreed that children and youngsters use smartphone applications to find information instead of the classic atlases, encyclopedias or dictionaries. The intelligent applications’ permanent evolution is pressing us to adapt to the technological changes and learn how to shape our geographical space that is no longer visualized through the classic globe. The future of sustainable Geosystems is inside the smartphones that our kids will use to creatively model the World’s environments, to generate, control and assess progress in a durable inquiry-based linkage between human values and new technology^63,64^.

The results suggest that prosperity tends to concentrate where it originated, being associated with young people, cooperative and effectively inter-connected communities. The remote traditional village houses from any world’s corner can easily become exquisite rural accommodations on the Airbnb platform^8^.

The remote Romanian village of Viscri has higher resilience than other rural communities because they were taught how to identify their Geosystem’s unique attributes and learned to reach and constantly assess the progress toward their sustainability development goals. Well connected to the Mihai Eminescu Trust (MET) international networks, the individual Viscri Geosystem is more adaptable to modern technological advances, conserving their traditional culture and promoting communal values^65^.

The role of indigenous peoples in the conservation and protection of ecosystems and natural resources has been noted. Accordingly, such people play a key role in safeguarding local ecosystems, conserving and protecting land and other natural resources^59^. Hence, the Ogiek would play a significant role in the protection and conservation of the Mau Forest ecosystem.

The need to conserve and protect the Mau ecosystem provides a strong case against allowing communities (including the Ogiek) to encroach into or settle in the Forest. It is noted that between the early 1990s (when encroachment and land allocation in the Forest intensified) and 2009, over 107,000 hectares (representing about a quarter of total forest cover) had been lost due to encroachment, illegal human settlement and forest extraction^66^. Although some portion of the Forest had been excised and degazetted in 2001, for purposes of settling the Ogiek and victims of the 1990 land classes in Kenya, it has been noted that the beneficiaries included non-deserving people such as Government officials and political leaders^66^.

Foreign energy inputs can stimulate the Geosystem’s transformation and development if the features relevant for its functionality are targeted. Carson and Carson (2018)^67^ argued that the immigrants are contributing to innovation in the small communities from northern Sweden. This research analysis show that the German investor knowledge input into the Romanian village of Cund helped the locals learn how to shape their Geosystems and thrive. Referring to the 2008 financial crash, Massey (2018)^6^ suggests that the distinctive use of stories and myths represent a type of egregore that can facilitate unusual resolutions from the policy makers and to incomprehensible behavior of the local authorities. Iorio and Corsale (2010)^68^ found that the Romanian policy makers are not taking into account the rural families’ development initiatives. The Danish investment in the village of Bagaciu collapsed due to the lack of support from regional authorities. There is a strong need for coherent governmental decisions to stimulate and protect the individual Geosystems‘ critical functionality for the long term.

The study finds that the donor-backed sustainable development projects do not have a persisting effect on communities because they usually focus on the short term^68^. According to Turnbull^69^, who invested in the Transylvanian village of Saschiz, a ten years minimum timeframe is necessary to ensure the sustainability of community projects. Currently, Saschiz’s dynamic Geosystem is in constant flux. The Transylvanian rural villages are emptied by economic migration while foreign private initiatives are focusing on the establishment of cooperative networks in the region. Saschiz Geosystem’s unique attributes, represented by the elder flower and the local fruits, were identified and valorized for the local community benefits^69^.

The results show that future sustainable Geosystems have to encourage innovation and constant learning in order to become more resilient and adaptive to change^12^. Currently, instead of using classic media (television, radio, newspapers) information for getting the best offer for supermarket vegetables, people should use the smartphone apps to find locally produced products and the rating systems to validate them. Moreover, they can use technology to learn how to grow their own vegetables and improve the harmony of their geographical space, ameliorating its virtual limitations^2^.

The analysis suggests that the indigenous collectives that identified their Geosystem’s authentic resources and honestly valorized them throughout all their actions, managed to integrate the social and environmental values to economic values and thrived. There is evidence that most indigenous communities prospered in their local geosystems through sustainable use of the local resources and hence advances in the use of the smartphone would go a long way in ensuring protection, conservation, and management of these resources for their benefits and the world in general.

## Conclusions

This article examined how the more localized Geosystems can become more resilient, emphasizing the importance of community-based social actions and the role of technological advances in the configuration process of our environments. When people deliberately come together for a collective ideal, a form of group mind is created. That represents an egregore which has to be preserved deliberately otherwise it will vanish. The easily apprehended Geomedia techniques can contribute to its maintenance and growing process so it can stimulate and support the realization of the community objectives.

The future Geosystem’s land management will integrate GIS data-based analytics with drones, self-driving measurement equipment, a diversity of sensors and smartphone applications. Automated geoponics has to be environmentally regenerative and include communities’ participation. The analytical thinking of local people will develop creative collaboration networks for the transference of information, products and potential collaborations.

The article reveals that the future pathway to sustainability combines the value of indigenous communities with a good knowledge of the last technological advances and the governmental policy makers’ support for the implementation and permanent assessment of global sustainable development objectives. Good examples of functional Geosystems can be found in countries such as the United States, Norway, Greece, China, Indonesia and Uzbekistan. Bolivia, Kenya, India and Romania who are struggling to fight poverty with adaptive development plans. Romania is blocked in terms of supportive development policy decisions by European Union membership restrictions and by the activities of environmental groups, which are more focused on wildlife protection than on the well- being of poor indigenous people.

According to this analysis, the World’s communities have to be aware of their whole dynamic Geosystems, which are in continual flux and learn how to enhance their abilities to model the sustainability processes and increase the quality of their life. In an effort to reclaim the Mau forest complex and protect further forest degradation within the complex, the Kenyan Government has been evicting people forcibly from the forest who had settled within and near the forest areas. The only option for the Ogiek indigenous community is to accept to be resettled by the Government outside the forest but nearby with easy access to the forest since the Ogieks are known to protect and live harmoniously with nature. This will ensure that no forest encroachments will happen either by the Ogieks or any other community. This will also allow for the replenishment of the various rivers from the forests which are at the verge of drying out due to human activities (agricultural activities and timber harvesting). Overall, when the Mau forest complex is protected and conserved then all the communities including the Ogieks will benefit immensely, thus the current government efforts must be supported at all costs.

The future of sustainability will not rely on the strongest community nor the most intelligent but it will rely on the one that is most adaptable to change. Thus, adaptation to change is the key to sustainable development, conservation and maintenance of ecosystems in the wake of increasing demands and uses of the diminishing natural resources.

Geosystem’s egregore preservation will fundament its adaptability to changes. People have to learn their living place history and actively maintain their ancient traditions for a strong collective mind formation. This will lead to the self-sustainable Geosystems development where the communities regulate and control the dynamic equilibrium state, always adapting to environmental and societal changes.

## References

[1] Paton, M. *Five Classics of Fengshui: Chinese Spiritual Geography in Historical and Environmental Perspective*. (Leiden-Boston, Brill, 2013).

[2] Yoon HK (2017). Indigenous ‘Folk‘ Geographical Ideas and Knowledge. Advances in Anthropology.

[3] Kelly, K. *Out of control: the new biology of machines*, *social systems and the economic world*, (Boston, Addison-Wesley, 1994).

[4] Martin, M. Taking on Being: Getting Beyond Postmodern Criticism, *The Midwest Quaterly* 51 (1) (2009).

[5] Dawkins, R. *Memes*, *Brief Candle in the Dark: My Life in Science*, (London, Bantam Press, 2015).

[6] Massey Andrew (2018). Persistent public management reform: an egregore of liberal authoritarianism?. Public Money & Management.

[7] Gupta SK, Negru R, Voda M (2018). The Indian Himalaya’s unique attributes: Hemkund Sahib and The Valley of Flowers. Geographia Technica.

[8] Ernawati NM, Torpan A, Voda M (2018). Geomedia role for mountain routes tourism development. Mesehe and Pisoiu Waterfall comparative study. *Geographia*. Technica.

[9] Voda Mihai, Torpan Adrian, Moldovan Lucian (2017). Wild Carpathia Future Development: From Illegal Deforestation to ORV Sustainable Recreation. Sustainability.

[10] Voda AI, Sarpe CA, Voda M (2018). Methods of maximum discharge computation in ungauged river basins. Review of procedures in Romania. Geographia Technica.

[11] Chambers D (2018). Tourism research: Beyond the imitation game. Tourism management Perspectives.

[12] Forum for the Future, Future of Sustainability 2018–Living in Nonlinear times. Available at, http://www.sustainablebrands.com/digital_learning/research_report/next_economy/future_sustainability_2018_living_nonlinear_times (2018).

[13] Shabanova Y (2017). Socio-Worldview Aspect of Energy Efficiency in the Context of the Postmaterialistic paradigm of Science, *Advanced Engineering*. Forum.

[14] Ravasz E, Barabasi A-L (2003). Hierarchical organization incomplex networks. Physical Review E.

[15] Lietaer, B. The Worgl Experiment: Austria (1932-1933). Available at, Curency Solutions for a Wiser World, https://www.lietaer.com/2010/03/the-worgl-experiment. (2010).

[16] Ryan C (2018). Future trends in tourism research- Looking back to look forward: The future of ‘Tourism Management Perspectives‘. Tourism Management Perspectives.

[17] Croft, D. Success in regenerative agriculture depends on collaboration. Available at, DIAGEO, http://www.sustainablebrands.com/digital_learning/research_report/next_economy/future_sustainability_2018_living_nonlinear_times (2018).

[18] Mac, I. Geomorfosfera și geomorfosistemele, (Presa Universitară Clujeană, Cluj Napoca, 1996).

[19] Park S (2017). & Carla Almeida Santos. Exploring the Tourist Experience: A Sequential Approach. Journal of Travel Research.

[20] Voda M, Montes YS (2018). Descending mountain routes future: the North Yungas and Fagaras Geosystem’s comparative study. Geographia Technica.

[21] Lapenta F (2011). Geomedia: on location-based media, the changing status of collective image production and the emergence of social navigation systems, *Visual*. Studies.

[22] ArcGIS Server, *Website of ArcGIS Server*. Environmental Systems Research Institute. Available at, www.esri.com/software/arcgis/arcgisserver/. (2017).

[23] Google Earth. *Google Earth Smartphone App*. Available at, www.google.com/earth/ (2017).

[24] Zheng Y-T, Zha Z-J, Chua T-S (2010). Research and applications on georeferenced multimedia: a survey. Multimedia Tools Applications.

[25] Stratigea A, Katsoni V (2015). A strategic policy scenario analysis framework for the sustainable tourist development of peripheral small island areas – the case of Lefkada-Greece Island. European Journal of Futures Research.

[26] Yanai, K., Yaegashi, K., & Qiu, B. Detecting cultural differences using consumer-generated geotagged photos. In: Proceedings of the 2nd international workshop on location and the web. ACM, New York, 1–4 (2009).

[27] Kütting, G. Thassos in *The Global Political Economy of the Environment and Tourism*, (London, Palgrave Macmillan, 2010).

[28] Liu L., Wolfson O. & Yin H. Extracting semantic location from outdoor positioning systems. In Proceedings of the IEEE International Conference on Mobile Data Management (2006).

[29] Inal C, Kocak O, Esen O, Bulbul S, Kizgut R (2017). Surveying and Mapping using Mobile Phone in Archaeological Settlements. *Geographia*. Technica.

[30] Arrow, K.J., Sen, A.K. & Suzumura, K. *Handbook of Social Choice and Welfare*, vol. 2, (Amsterdam, North-Holland, 2011).

[31] Echtner CM, Brent Ritchie JR (2003). The meaning and measurement of destination image. The Journal of Tourism Studies.

[32] Valentino-DeVries, J. Using flickr photos as a travel guide. Wall Street J July 23. Available at, http://blogs.wsj.com/digits/2010/07/23/using-flickr-photos-as-a-travel-guide/ (2010).

[33] Lai K, Li XR (2016). Tourism Destination Image: Conceptual Problems and Definitional Solutions. Journal of Travel Research.

[34] Asero V, Gozzo S, Tomaselli V (2016). Building Tourism Networks through Tourist Mobility. Journal of Travel Research.

[35] Cherifi B, Smith A, Maitland R, Stevenson N (2014). Destination images of non-visitors. Annals of Tourism Research.

[36] Larson LR, Poudyal NC (2012). Developing sustainable tourism through adaptive resource management: a case study of Machu Picchu, Peru. Journal of Sustainable Tourism.

[37] Barbieri C, Xu S, Gil-Arroyo C, Rozier Rich S (2016). Agritourism, Farm Visit, or…? A Branding Assessment for Recreation on Farms. Journal of Travel Research.

[38] Airbnb. Airbnb platform. Available at, http://www.airbnb.com (2018).

[39] Google Maps. *Google Maps Smartphone App*. Available at, www.google.com/maps/ (2018).

[40] Delaforge, G. The Templar Tradition: Yesterday and Today, *Gnosis Magazine*, **6** (1987).

[41] Hwang D, Stewart WP (2017). Social Capital and Collective Action in Rural Tourism. Journal of Travel Research.

[42] Dawkins, R. *The selfish gene*, (New York, Oxford University Press, 1976).

[43] House, J. Redefining sustainability: A structural approach to sustainable tourism in *Tourism and sustainability principle to practice* (ed.Stabler, M. J.) 89–104 (Oxon, UK, Biddles ltd, 1997).

[44] Rossbach, S. *Feng Shui: The Chinese art of placement*. (New York, E.P. Dutton, 1983).

[45] Stokols D (1990). Instrumental and spiritual views of people-environment relations. American Psychologist.

[46] Weng T (2008). Application Internet Multimedia on Region Travel Route Information Establishment. WSEAS Transactions on Computers.

[47] Lin CT (2012). Classification of the high-mountain coniferous in Taiwan. Folia Geobotanica.

[48] Vervoorn A (1990). Cultural Strata of Hua Shan, the Holy Peak of the West,. Monumenta Serica-Journal of Oriental Studies.

[49] Sutawa GK (2012). Issues on Bali tourism development and community empowerment to support sustainable tourism development. Procedia Economics and Finance.

[50] Catanoiu, S. The Carpathian Mountains, a realm of Sacred Natural Sites, in Mallarach, J.-M., Papayannis, T. and Väisänen, R. (eds) The Diversity of Sacred Lands in Europe: Proceedings from *The Third Workshop of the Delos Initiative* – Inari/Aanaar 2010. Gland, Switzerland: IUCN and Vantaa, Finland: Metsähallitus Natural Heritage Services (2012).

[51] Comanescu L, Nedelea A, Dobre R (2013). The geotouristic map- between theory and practical use. Case Study: the central sector of the Bucegi Mountains (Romania), Geojournal of Tourism and Geosites.

[52] Negi, S.S. *Himalayan Rivers*, *Lakes*, *and Glaciers*. (New Delhi, Indus Publishing, 1991).

[53] Singh RB, Mal S, Kala CPJ (2009). Community responses to mountain tourism: A case in Bhyundar Valley, Indian Himalaya. Journal of Mountain Science.

[54] European Commission. Natura 2000 standard data form: explanatory notes. (European Commission, Brussels, 2000).

[55] Votsi NE (2012). Road effects on habitat richness of the Greek Natura 2000. network. Nat Conserv.

[56] Towett, J.K. *Ogiek Land Cases and Historical Injustices 1902-2004*, (Ogiek Welfare Council, 2004).

[57] Minority Rights International. Huge victory for Kenya’s Ogiek as African Court sets major precedent for indigenous peoples’ land rights. Available at, https://minorityrights.org/2017/05/26/huge-victory-kenyas-ogiek-african-court-sets-major-precedent-indigenous-peoples-land-rights/ (2018).

[58] Wachira, M.W. Vindicating Indigenous Peoples’ Land Rights in Kenya, Unpublished thesis submitted for the degree, Doctor of Laws, University of Pretoria. (2008).

[59] African Court of Human and Peoples Rights. African Commission on Human and Peoples’ Rights Versus Republic of Kenya, Application No. 006/2012, Arusha Tanzania (2017).

[60] Swart, R. E. Monitoring 40 Years of Land Use Change in the Mau Forest Complex, Kenya: a Land Use Change Driver Analysis, in a thesis submitted in partial fulfillment of the degree of Master of Science at Wageningen University and Research, The Netherlands (2016).

[61] Sutton K, Fahmi W (2002). The rehabilitation of Old Cairo. Habitat International.

[62] Airey, D., & Shakley, M. *Chapter 2- Bukhara* (*Uzbekistan*)*: A former oasis town on the Silk Road*, in Visitor Management: (Butterworth-Heinmann, 1998).

[63] Jarvis CH, Kraftl P, Dickie J (2017). Re) Connecting spatial literacy with children’s geographies: GPS, Google Earth and children’s everyday lives. Geoforum.

[64] Sjøberg Svein (2017). Pisa testing. Europhysics News.

[65] Iorio M, Corsale A (2014). Community-based tourism and networking: Viscri, Romania. Journal of Sustainable Tourism.

[66] Government of Kenya. Report of the Prime Minister’s Task Force on the Conservation of the Mau Forests Complex, *Unpublished* (2009).

[67] Carson, D.A., Carson, D.B. International lifestyle immigrants and their contributions to rural tourism innovation: Experiences from Sweden’s far north. *Journal of Rural Studies*, In Press. (2018)

[68] Iorio M, Corsale A (2010). Rural tourism and livelihood strategies in Romania. Journal of Rural Studies.

[69] Financial Times, From Oxfordshire to Transylvania: cordial relations. Available at, www.ft.com (2018).

